# MRI-based inter- and intrafraction motion analysis of the pancreatic tail and spleen as preparation for adaptive MRI-guided radiotherapy in neuroblastoma

**DOI:** 10.1186/s13014-023-02347-9

**Published:** 2023-10-02

**Authors:** Fasco Van Ommen, Gaelle A.T. le Quellenec, Mirjam E. Willemsen-Bosman, Max M. van Noesel, Marry M. van den Heuvel-Eibrink, Enrica Seravalli, Petra S. Kroon, Geert O. Janssens

**Affiliations:** 1 Department of Radiation Oncology, University Medical Center Utrecht, Heidelberglaan 100, Utrecht, 3584 CX The Netherlands; 2 Department of Radiation Oncology, Institut de Cancérologie de l’Ouest, Nantes, France; 3 Princess Máxima Center for Pediatric Oncology, Utrecht, The Netherlands

**Keywords:** Neuroblastoma, Pediatric radiotherapy, Interfraction motion, Intrafraction motion, MRI-guided radiotherapy

## Abstract

**Background:**

In pediatric radiotherapy treatment planning of abdominal tumors, dose constraints to the pancreatic tail/spleen are applied to reduce late toxicity. In this study, an analysis of inter- and intrafraction motion of the pancreatic tail/spleen is performed to estimate the potential benefits of online MRI-guided radiotherapy (MRgRT).

**Materials and methods:**

Ten randomly selected neuroblastoma patients (median age: 3.4 years), irradiated with intensity-modulated arc therapy at our department (prescription dose: 21.6/1.8 Gy), were retrospectively evaluated for inter- and intrafraction motion of the pancreatic tail/spleen. Three follow-up MRIs (T2- and T1-weighted ± gadolinium) were rigidly registered to a planning CT (pCT), on the vertebrae around the target volume. The pancreatic tail/spleen were delineated on all MRIs and pCT. Interfraction motion was defined as a center of gravity change between pCT and T2-weighted images in left-right (LR), anterior-posterior (AP) and cranial-caudal (CC) direction. For intrafraction motion analysis, organ position on T1-weighted ± gadolinium was compared to T2-weighted. The clinical radiation plan was used to estimate the dose received by the pancreatic tail/spleen for each position.

**Results:**

The median (IQR) interfraction motion was minimal in LR/AP, and largest in CC direction; pancreatic tail 2.5 mm (8.9), and spleen 0.9 mm (3.9). Intrafraction motion was smaller, but showed a similar motion pattern (pancreatic tail, CC: 0.4 mm (1.6); spleen, CC: 0.9 mm (2.8)). The differences of Dmean associated with inter- and intrafraction motions ranged from − 3.5 to 5.8 Gy for the pancreatic tail and − 1.2 to 3.0 Gy for the spleen. In 6 out of 10 patients, movements of the pancreatic tail and spleen were highlighted as potentially clinically significant because of ≥ 1 Gy dose constraint violation.

**Conclusion:**

Inter- and intrafraction organ motion results into unexpected constrain violations in 60% of a randomly selected neuroblastoma cohort, supporting further prospective exploration of MRgRT.

## Background

Most pediatric patients undergoing radiotherapy to the upper abdominal region are diagnosed with a neuroblastoma or a renal tumor. Survivors are at risk of long-term toxicity with radiotherapy being a major determinant [1–8]. A number of late effects like vertebral growth impairment or vascular damage are inherent to the location of the target volume and therefore unavoidable by modern radiotherapy [9, 10]. Meanwhile, radiotherapy doses to a number of organs at risk (OARs) like the pancreas, spleen or intestines can be significantly reduced using state-of-the art approaches [5, 6, 8, 11–15]. The risk of diabetes due to radiotherapy on the pancreatic tail has a linear dose-response relationship with a threshold around 10 Gy. The cumulative incidence for diabetes by age 45 due to radiation on the pancreas is 4.3% for 1.0–9.9 Gy, 12.7% for 10.0–19.9 Gy and 25.7% for 20.0–29.9 Gy [11, 13]. For the spleen, a more binary threshold is observed. Nowadays, patients receiving a mean spleen dose above 10 Gy are recommended for antibiotic prophylaxis and vaccination to reduce the risk of late infection-related mortality [14]. The cumulative incidence of infection-related late mortality at 35 years after splenic radiation is 0.4% for 0.1–9.9 Gy, 1.1% for 10.0–19.9 Gy and 1.3% for 20.0–29.9 Gy [6].

Long-term toxicity of OARs prone to motion in between or during radiotherapy sessions may be reduced by exploiting the benefits of an online adaptive radiotherapy workflow. The latter utilizes daily imaging not only to correct for patient positioning, but also for daily anatomical changes. The new anatomy is used to re-optimize the treatment plan before every fraction [16]. A potential candidate for an online adaptive radiotherapy workflow is magnetic resonance guided radiotherapy (MRgRT) [17, 18]. For target volumes located in the upper abdominal region, MRI has superior soft-tissue contrast over CBCT-scans and allows visualization of the daily anatomy. An additional major benefit of MRgRT is the possibility for beam-on imaging. During dose delivery MR images can be acquired, which allows tumor and OAR motion monitoring. These images could be used for gating or potentially tumor tracking [19].

The potential role of MRgRT in pediatric radiotherapy has been identified based on a survey among (future) users [20] and has demonstrated dosimetric benefit in a plan comparison study for pediatric renal tumors [21]. For pediatric patients with a neuroblastoma or a renal tumor, the abilities of MRgRT to re-optimize a treatment plan to the daily anatomy may reduce radiation dose to the pancreatic tail and spleen, which might be beneficial for the long-term burden.

The purpose of this study is to evaluate the inter- and intrafraction motion of the pancreatic tail and spleen and to estimate the dose deviations from the original treatment plan due to these positional changes, including the number of dose constraint violations.

## Materials and methods

### Patient population

A total of ten randomly selected pediatric patients with a neuroblastoma originating from the upper abdomen and treated, between May 2019 and September 2021 within the department of radiation oncology Utrecht, were included for this analysis. Candidate patients had a 4DCT- and MRI-scan in radiotherapy position, as well as three additional MRI exams performed within 12 months post-radiotherapy. For all MRI exams T2- and T1-weighted sequences with and without intravenous gadolinium were required. If MRI-artefacts were visible in any of the scans, the entire MRI exam was omitted. The retrospective analysis was approved by the local ethics committee (Institutional Review Board approval number: WAG/mb/500,028).

### Imaging data

For each patient, a pre-treatment 4DCT-scan (pCT; average CT of ten phases, helical acquisition and reconstructed to 2 mm slice thickness) was acquired for treatment planning as well as an MRI-scan (T1/T2-weighted with 3 mm slice thickness). The latter was used for delineation purposes only. The contours of the pancreatic tail and spleen on the pCT were considered as the reference position.

**Fig. 1:**
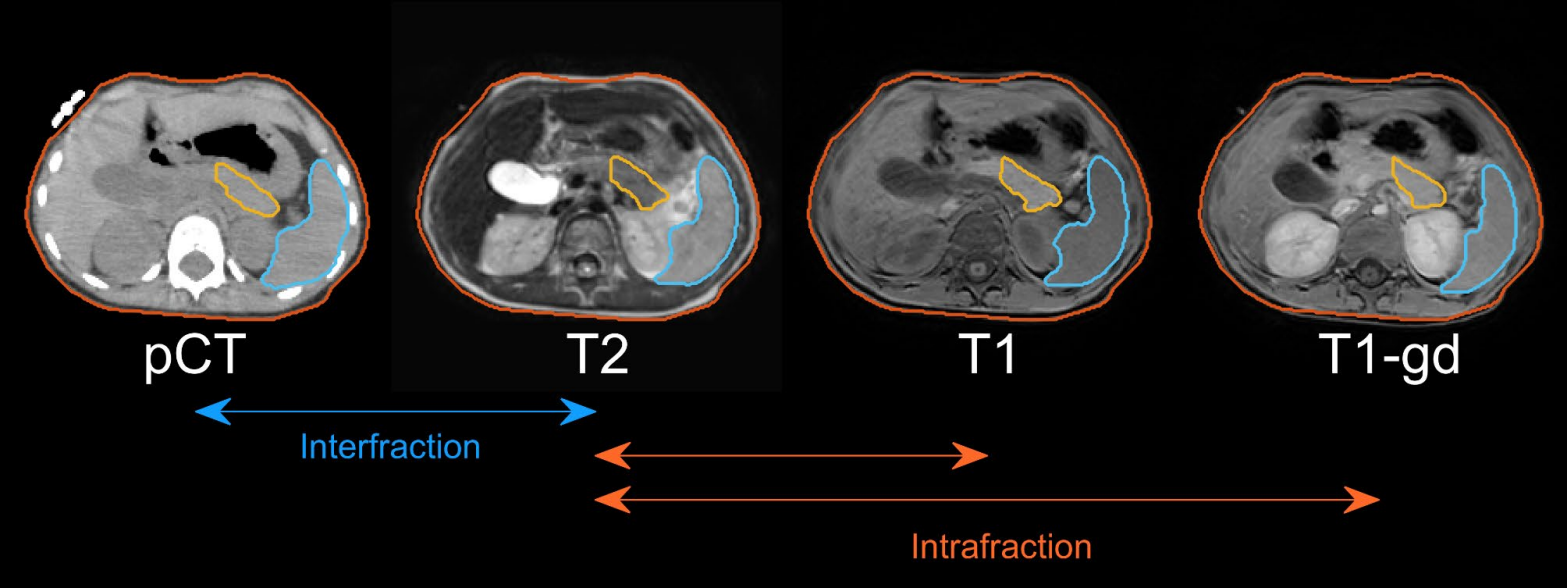
Example of registered T2, T1 and T1 with gadolinium (T1-gd) to the planning CT (pCT) with the pancreatic tail (orange) and spleen (blue) delineated. Interfraction motion is defined as positional changes between pCT and T2, whereas intrafraction motion is defined as positional changes between T2 and T1/T1-gd

Post-radiotherapy, MRI-scans are repeated before onset, during and after immunotherapy to monitor tumor response or early disease progression according to institutional protocol. For all ten patients, free-breathing T2 and T1 sequences with/without gadolinium acquired during the three MRI exams were co-registered to the vertebrae on the pCT at the level of the primary tumor, pancreas and spleen using rigid registration. The registration used a mutual information method. Following registration, the pancreatic tail and spleen were delineated on all nine MRI sequences by an experienced radiation oncologist [GLQ, supervised by GJ] (Fig. 1).

### Motion

To assess the contribution of respiratory motion on the pCT-scan, the 4DCT was evaluated using the time-averaged mid-position algorithm [22]. The mid-position CT is calculated from the 4DCT, but also provides information about the amplitude of respiratory motion. The position of the pancreatic tail and spleen was defined by the center of gravity (COG). Motion was evaluated in three directions (cranial-caudal (CC), left-right (LR) and anterior-posterior (AP)). Intrafraction motion was evaluated by comparing the position of the pancreatic tail and spleen of the second and last sequence to the first acquired during the same MRI exam (60 measurements per organ: 10 patients x 3 MRI exams x 2 sequences). Interfraction motion of the pancreatic tail and spleen was evaluated by comparing the position of these organs on the T2-weighted MRI of each MRI exam to the position on the reference (30 measurements per organ: 10 patients x 3 MRI exams).

### Analysis

The respiratory, inter- and intrafraction motions were visualized using boxplots. To estimate the radiotherapy dose to the pancreatic tail and spleen delineated on the MRI exams, the dose distribution used for clinical treatment was overlaid and the mean dose to the volumes defined on MRI was determined. In daily practice at our department, a mean dose constraint of 10 Gy is applied for the pancreatic tail and spleen, respectively corresponding to an increased risk of diabetes mellitus and late infection-related mortality [6, 11, 14, 23]. Fulfillment of the dose constraints of the pancreatic tail or spleen was considered to be of ‘potential clinical relevance’ and therefore of potential benefit of daily replanning and gating. If the dose on the pCT was below the mean dose constraint but exceeded on the MRI, it was highlighted.

### Statistical analysis

The analysis was descriptive in which the range of the respiratory, intra- and interfraction motion and the dose differences were reported.

## Results

### Patient, tumor, and treatment characteristics

Patient, tumor and treatment characteristics are demonstrated in Table 1. Six patients were treated for a left-sided neuroblastoma. The median age at treatment was 3.4 years old (range: 1.1–8.6). Six out of ten patients were under sedation (anesthesia) during treatment preparation and treatment. All patients were sedated during follow-up MRI.

**Table 1:** Patient, tumor and treatment characteristics

	Number
**Patient characteristics**	
Gender	
Male	6
Female	4
Age at diagnosis (years)	
Median	3.4
Range	1.1–8.6
**Tumor characteristics**	
Type	
Neuroblastoma	10
Primary tumor location	
Adrenal gland	9
Paravertebral	1
Side	
Left	6
Right	4
**Treatment characteristics**	
Prescribed dose	
21.6/1.8 Gy	10
Anesthesia	
Yes	6
No	4

### Respiratory motion

**Fig. 2:**
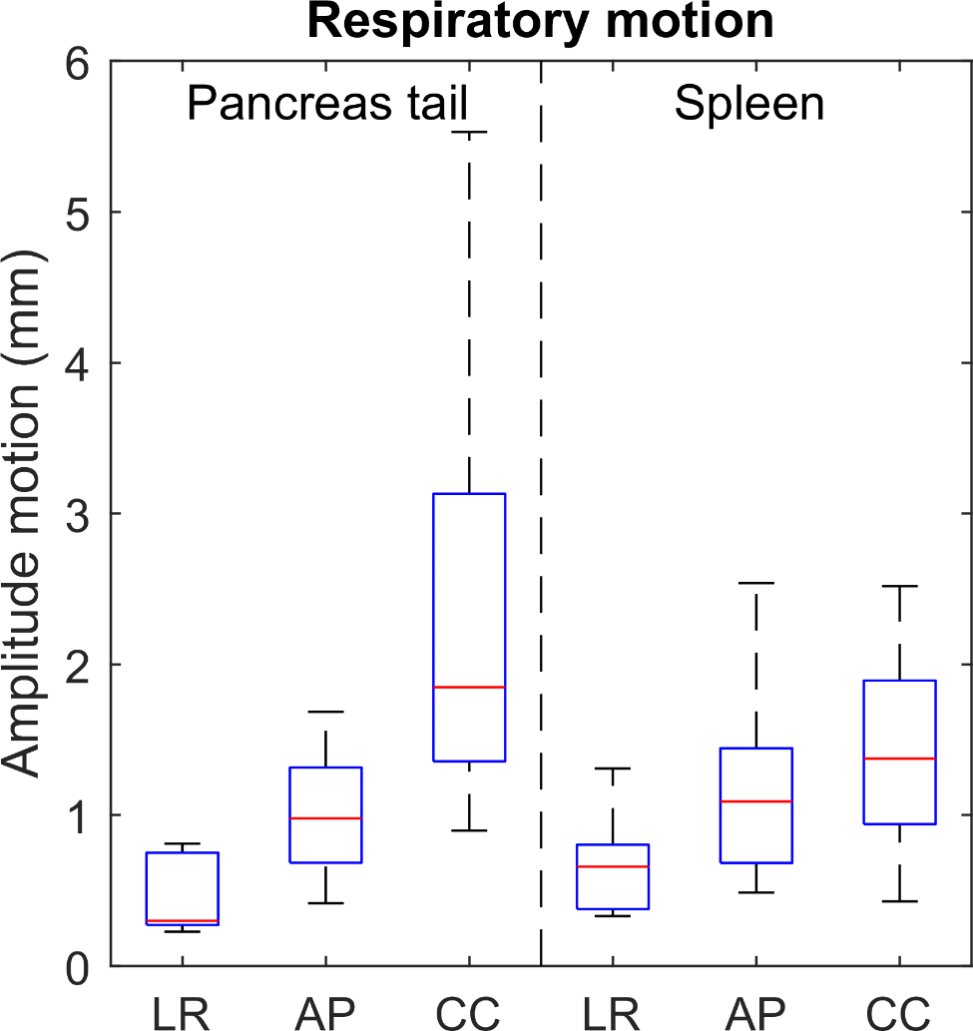
Amplitude of respiratory motion (mm) for the pancreatic tail and spleen, calculated on the 4DCT, is shown as a function of the three directions left-right (LR), anterior-posterior (AP) and cranial-caudal (CC)

The respiratory motion of the pancreatic tail and spleen is predominantly in the CC direction (Fig. 2). The amplitude of this motion is larger for the pancreatic tail (median amplitude: 1.8 mm) compared to the spleen (median amplitude: 1.4 mm), whereas the motion in LR and AP directions is similar for both organs.

### Intrafraction motion

**Fig. 3:**
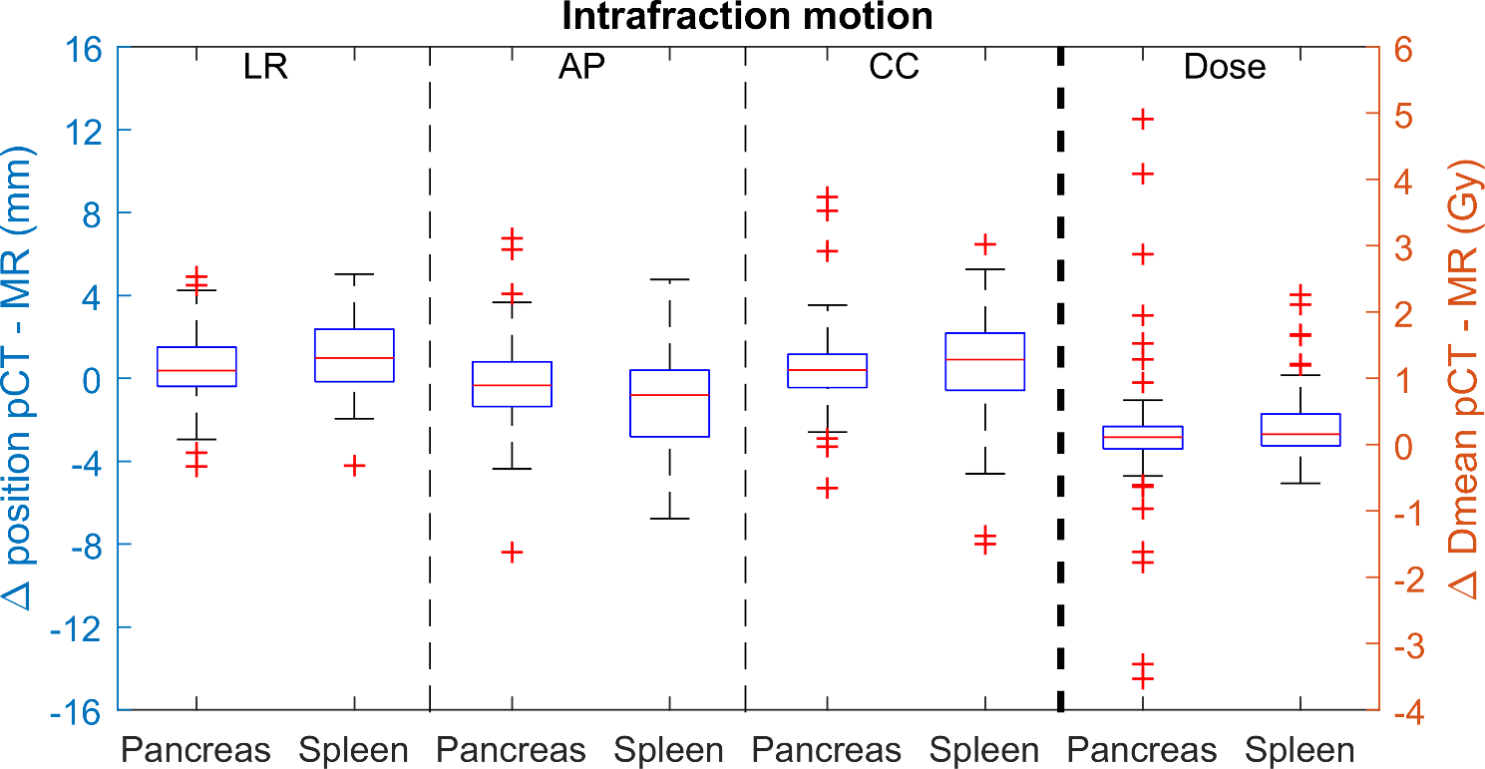
Intrafraction motion (mm) of the pancreatic tail (Pancreas) and spleen for ten patients having six intrafraction movements. Motion is shown in three directions (left-right (LR), anterior-posterior (AP) and cranial-caudal (CC)). The dose difference (Gy) between the contours delineated on the pCT and the different MRI-scans are shown on the right

For the pancreatic tail, the intrafraction motion ranged from − 4.3 to 4.9 mm for LR, -8.4 to 6.8 mm for AP and − 5.3 to 8.8 mm for CC (Fig. 3). For the spleen, LR ranged from − 4.2 to 5.0 mm, AP from − 6.8 to 4.8 mm and CC from − 8.0 to 6.5 mm.

The dose differences for both pancreatic tail and spleen between pCT and MRI-exams (60 measurements per organ) are minimal but show some large outliers (Fig. 3). These outliers are predominantly observed in patients with left-sided tumors. In addition, the dose differences are smaller for the spleen compared to the pancreatic tail and have less excessive outliers (range − 3.5 to 4.9 Gy for the pancreatic tail; -0.6 to 2.3 Gy for the spleen). For the pancreatic tail and spleen, respectively 12 and 24 out of 60 intrafraction movements, all observed in 6 patients, were highlighted as potentially clinically significant because of a dose constraint violation (Table 2).

**Table 2:** Dose constraint violations of the pancreatic tail and spleen for intra- and interfraction motion, including the number of unexpected mean dose violations above 10 Gy

			pCT	Interfraction motion			Intrafraction motion		
Patient	Side	OAR	D_mean_ (Gy)	D_mean_ range (Gy)	Fractions D_mean_ < 10 Gy (N)	Unexpected dose violation (N)	D_mean_ range (Gy)	Fractions D_mean_ < 10 Gy (N)	Unexpected dose violation (N)
1	Right	Pancreas tail	6.3	6.3–6.7	3/3		6.4–7.1	6/6	
		Spleen	2.3	2.3–2.5	3/3		2.5–2.9	6/6	
2	Left	Pancreas tail	21.0	21.0–21.2	0/3		21.0–21.3	0/6	
		Spleen	7.5	6.7–10.1	2/3	1/3	6.7–11.3	3/6	3/6
3	Left	Pancreas tail	6.9	5.7–10.4	2/3	1/3	7.0–11.4	4/6	2/6
		Spleen	7.5	7.9–8.8	3/3		7.6–9.2	6/6	
4	Right	Pancreas tail	3.8	0.6–6.2	3/3		0.5–5.9	6/6	
		Spleen	0.2	0.1–0.5	3/3		0.2–0.5	6/6	
5	Left	Pancreas tail	13.8	12.9–13.1	0/3		12.9–15.0	0/6	
		Spleen	7.0	6.6–7.8	3/3		7.0–7.9	6/6	
6	Left	Pancreas tail	20.8	20.5–20.9	0/3		18.9–21.3	0/6	
		Spleen	8.7	9.8–10.5	2/3	1/3	9.7–11.1	2/6	4/6
7	Right	Pancreas tail	9.4	9.4–11.7	2/3	1/3	9.4–14.3	2/6	4/6
		Spleen	2.6	2.7–3.0	3/3		2.6–3.0	6/6	
8	Left	Pancreas tail	11.8	15.7–17.6	0/3		12.3–17.7	0/6	
		Spleen	9.3	8.4–8.7	3/3		9.3–10.7	3/6	3/6
9	Right	Pancreas tail	3.4	2.2–4.9	3/3		2.1–5.2	6/6	
		Spleen	3.0	2.9–4.3	3/3		2.9–4.5	6/6	
10	Left	Pancreas tail	20.9	19.9–21.0	0/3		19.9–21.2	0/6	
		Spleen	9.8	9.7–10.6	1/3	2/3	9.5–10.4	1/6	5/6

### Interfraction motion

**Fig. 4:**
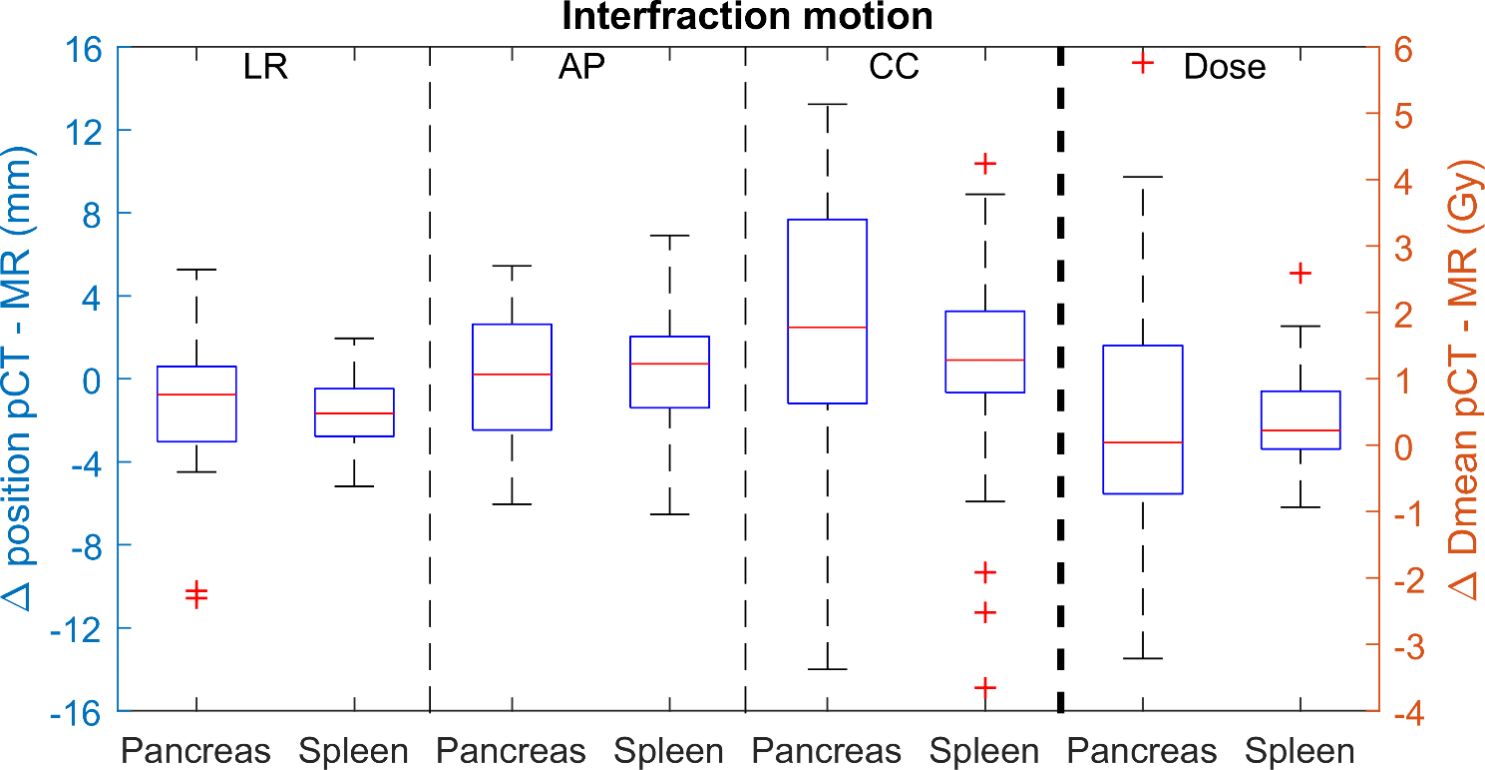
Interfraction motion (mm) of the pancreatic tail (Pancreas) and spleen for ten patients having three interfraction movements. Motion is shown in three directions (left-right (LR), anterior-posterior (AP) and cranial-caudal (CC)). The dose difference (Gy) between the contours delineated on pCT and the different MRI-exams are shown on the right

The interfraction motion of both the pancreatic tail and spleen shows a pattern similar to the intrafraction motion (Fig. 4). The median LR motion is smallest (range pancreatic tail: -10.6 to 5.3 mm and range spleen: -5.2 to 2.0 mm) followed by the AP (range − 6.0 to 5.4 mm for the pancreas tail and range − 6.5 to 6.9 mm for the spleen) and CC (pancreas tail ranged from − 14.0 to 13.2 mm and the spleen ranged from − 14.9 to 10.4 mm) motion.

After co-registration of the MRI to the vertebrae on the pCT, a large range in dose difference between pCT and MRI for the pancreatic tail (-3.2 to 5.8 Gy) is observed. For the spleen this range was found to be smaller (-1.2 to 3.0 Gy). Interfraction motion has little or no impact on the dose to the pancreatic tail and spleen in right-sided tumors. For the pancreatic tail and spleen, respectively 6 and 9 out of 30 fractions, all observed in 5 patients, resulted in a potentially clinically significant dose constraint violation (Table 2).

## Discussion

In this cohort of pediatric patients with a primary neuroblastoma in the upper abdominal region, the respiratory, intra- and interfraction motion of the pancreatic tail and spleen was assessed. In addition, dose differences on the pancreatic tail and spleen due to these movements have been presented, using upfront dose overlay. The impact of motion on dose distribution for the pancreatic tail and spleen was obvious for left-sided target volumes and only minimal for right-sided targets. In six out of ten patients at least one of the dose constraints was exceeded unexpectedly due to inter- or intrafraction motion.

In general, interfraction motion (due to day-to-day differences, such as differences in bowel or bladder filling) is largest followed by the intrafraction motion (due to drift, respiration or sudden movements), which is in accordance with prior CT/CBCT based retrospective analyses [24–26]. To our knowledge, there has not been a study investigating the motion of the pancreatic tail in pediatric patients. The larger interfraction motion compared to intrafraction motion suggests that interfraction motion is not a snapshot of positional changes due to breathing, but also other anatomical changes, for which daily replanning would be a useful tool.

The wide range of doses to the pancreatic tail and spleen observed when taking into account interfraction motion demonstrates that a single capture of the anatomy, as routinely used, can over- or underestimate the actual dose. The latter incorrectly informs patients about their increased risk of diabetes or lifetime need for antibiotics and vaccination when the organ constraints are violated [13, 14]. In addition, the possibility of anticipating on the daily anatomy changes with daily replanning can even have a larger dosimetric benefit for both organs. The dose differences due to intrafraction motion are generally smaller than those due to interfraction motion, but still in cases where a drift of an organ is visible, motion management during treatment available with MRgRT can reduce dosage to OARs. Nevertheless, the real benefit due to replanning and/or motion management during the whole course of radiotherapy needs to be evaluated extensively in a prospective way. For now, the largest clinical benefit of MRgRT is hypothesized for patients which have a minimal exceedance of the dose constraint on the pancreatic tail and/or spleen, which by replanning alone could be overcome and reduced to a mean radiation dose level below 10 Gy.

In this study, we mainly focused on the use of MRI to visualize the daily anatomy, whereas the same could be achieved using high quality CBCT imaging, as already shown in adults [27, 28]. However, the small amount of intra-abdominal fatty tissue in young pediatric patients limits optimal visualization of the pancreatic tail and other abdominal organs. Hence, the unique value of soft-tissue contrast on MRI compared to CBCT image quality is the main rationale to investigate/explore the role of MRgRT for an online adaptive radiotherapy approach in pediatric patients with neuroblastoma and renal tumors.

In recent years, other alternatives for treating neuroblastoma have been proposed [29, 30]. Especially, proton beam therapy has been increasingly recommended for pediatric patients with neuroblastoma, even though this irradiation type can suffer dosimetric degradation from gastrointestinal air and tumor location and still requires large cohort studies to prove oncological benefit compared to state-of-the art photon therapy [31, 32]. Secondly, image guidance for proton therapy does not allow online dose recalculation and so limiting the technique to a static image in which inter- and intrafraction motion of the pancreatic tail and spleen is not compensated. The potential benefit of proton therapy mainly concerns the possibility to reduce a low-dose bath to the surrounding tissues to reduce the risk of secondary tumors. However, recent publications demonstrate the very low risk of second malignant neoplasm after abdominal irradiation for neuroblastoma or Wilms tumors, and with most neoplasms either unrelated to the radiotherapy beams, or in the high-dose area and therefore unavoidable by technique [8, 33, 34].

This analysis has a number of limitations. First, the post radiotherapy MRI-scans are not in treatment position, which makes a rigid registration between pCT and MRI more challenging. However, the inter- and intrafraction measured motion of the spleen, is in accordance with literature on CBCT’s and CT’s where patients were positioned in radiation position [24, 25]. This makes us believe that the chosen approach is appropriate for this analysis. Secondly, the follow-up MRIs are acquired within 10 months after finishing treatment, meaning that growth could cause additional motion compared to the pCT. For this reason we looked at the weight, height and diameter of these children during radiotherapy and at final MRI follow-up and did not see large differences (maximum difference weight: 2.5 kg and maximum height difference: 4.0 cm). The maximum diameter changes of the last MRI at follow-up compared to the last CBCT during treatment were similar (MRI; LR: 5.7 mm and AP: 4.0 mm and CBCT; LR: 7.4 mm and AP: 6.9 mm), suggesting no significant growth. Thirdly, the assumption is made that the dose distribution does not change when using a slightly different patient anatomy. This allows comparison of the radiation dose to the pancreatic tail and spleen without recalculation. In a future prospective pilot study, radiation dose will be evaluated on daily MRIs.

In conclusion, for pediatric patients with a neuroblastoma in the upper abdominal region online adaptive MRI-guided radiotherapy may have the potential to reduce the dose to the pancreatic tail and/or spleen. The approach is most promising in left-sided target volumes adjacent to the pancreatic tail and spleen receiving a prescribed dose with limited exceedance of the dose constraints. A prospective investigation of MRI-guided radiotherapy is required to confirm whether the risk of diabetes mellitus or an indication for spleen prophylaxis is better discussed with parents before or after the end of radiotherapy.

## Data Availability

The data used and generated in this work may be available under ethical and data protection considerations upon request to the leading institution on an individual basis.

## Abbreviations

MRgRT: MRI-guided radiotherapy
pCT: Planning CT
LR: Left - Right
AP: Anterior - Posterior
CC: Cranial - Caudal
IQR: Interquartile range
OARs: Organs at risk
COG: Center of gravity
